# A pan-transcriptome analysis shows that disease resistance genes have undergone more selection pressure during barley domestication

**DOI:** 10.1186/s12864-018-5357-7

**Published:** 2019-01-07

**Authors:** Yanling Ma, Miao Liu, Jiri Stiller, Chunji Liu

**Affiliations:** 1 CSIRO Agriculture & Food, 306 Carmody Road, St Lucia, QLD 4067 Australia; 2 Crop Research Institute of Sichuan Academy of Agricultural Sciences, Chengdu, 610066 China

**Keywords:** Barley, Pan-transcriptome, Novel transcripts, Wild, Cultivated, Resistance

## Abstract

**Background:**

It has become clear in recent years that many genes in a given species may not be found in a single genotype thus using sequences from a single genotype as reference may not be adequate for various applications.

**Results:**

In this study we constructed a pan-transcriptome for barley by de novo assembling 288 sets of RNA-seq data from 32 cultivated barley genotypes and 31 wild barley genotypes. The pan-transcriptome consists of 756,632 transcripts with an average N50 length of 1240 bp. Of these, 289,697 (38.2%) were not found in the genome of the international reference genotype Morex. The novel transcripts are enriched with genes associated with responses to different stresses and stimuli. At the pan-transcriptome level, genotypes of wild barley have a higher proportion of disease resistance genes than cultivated ones.

**Conclusions:**

We demonstrate that the use of the pan-transcriptome dramatically improved the efficiency in detecting variation in barley. Analysing the pan-transcriptome also found that, compared with those in other categories, disease resistance genes have gone through stronger selective pressures during domestication.

**Electronic supplementary material:**

The online version of this article (10.1186/s12864-018-5357-7) contains supplementary material, which is available to authorized users.

## Background

The phenomenon that an individual contains only a proportion of the genes in a given species was initially noticed in microbes [1]. This phenomenon led to the concept of pan-genome which consists of core and dispensable genomes [2, 3]. The core component contains genes shared by all individuals of a given species and the remainder belong to the dispensable component [3]. Reports on pan-genome for major crop species appeared only in recent years. Similar to that observed in various microbes, large proportions of genes in different crop species have also been found to be dispensable. For example, dispensable genes account for about 20% of the genomes in soybean (*Glycine soja*) [4] and *Brassica oleracea* [5], 36% in bread wheat (*Triticum aestivum*) [6, 7], 50% in maize (*Zea mays*) [8, 9] and 43% in rice (*Oryza sativa* L.) [10].

Cultivated barley (*Hordeum vulgare ssp. vulgare*), derived from its wild progenitor *H. vulgare* spp. *spontaneum*, is an ancient crop that was domesticated about 10,000 years ago in the Fertile Crescent. It is an important crop growing in highly diverse environments, and has been widely used as human food, animal feed and for fermented and distilled beverages [11]. Barley is a diploid and inbreeding species with a large haploid genome of 5.1 gigabases (Gb) and about 80% of the genome is characterised by repetitive elements and large pericentromeric regions that are virtually devoid of meiotic recombination [11, 12]. Even with the rapid progress in sequencing technique and capacity, it is still challenging to generate a high quality genome assembly for species like barley. After more than a decade of dedicated efforts, an international team consisting of over 70 researchers have successfully sequenced the complete genome of barley based on the cultivar Morex [12]. It seems unlikely that high quality genome assemblies for a large number of barley genotypes can be obtained in the near future. Thus a high quality pan-genome of barley may have to wait. However, capturing the majority of the expressed genes in a barley genotype is now relatively easy and inexpensive. Transcriptome profiling based on RNA-seq has in recent years become the technique of choice in capturing different species of transcripts including mRNAs, non-coding RNAs and small RNAs [13]. RNA-seq data from a wide range of studies with different objectives are now available for not only cultivated but also wild barley genotypes. They include studies varying from stress tolerance [14–18] to domestication [19, 20] and plant development [21, 22]. The availability of such a large quantity of RNA-seq datasets provides a good opportunity to construct a pan-transcriptome to capture most of expressed genes in barley genome.

## Methods

### Collection of RNA-seq data

A total of 288 sets of RNA-seq data from 63 barley genotypes were collected for this study. Of these, 234 were derived from 32 cultivated genotypes (*Hordeum vulgare ssp. vulgare*) and 54 from 31 wild accessions (*Hordeum vulgare ssp. spontaneum*) (Additional file 1: Table S1). The data consisted of a total of 6,321,262,514 reads downloaded from the EMBL (European Molecular Biology Lab)/EBI (European Bioinformatics Institute)-European Nucleotide Archive (ENA) database and the National Center for Biotechnology Information (NCBI) Short Sequence Read Archive (SRA) database. The sequences were obtained from a wide range of studies on different environmental factors and stress treatments including low temperature (vernalisation) [23, 24], photoperiod [25], drought [15], salinity [14, 17, 26], heat stress [18], disease infection [NCBI-GEO (Gene Expression Omnibus) accession GSE83676] and excessive nutrients [16]. Some of the sequences were obtained from studies on domestication [19, 20], tissue development ([21, 22], NCBI-GEO accession GSE87377) and whole genome sequencing in barley [11].

### Transcriptome reconstruction

Trimming and filtering of raw RNA reads were performed with SolexaQA++ software [27]. As the average spot lengths (AvgSpotLen) of different RNA-seq datasets were not the same, different filtering standards were applied to exclude low quality reads (Additional file 2: Table S2). Using Trinity (version 2.0.6) [28] with K-mers = 25, cleaned RNA-seq reads were pooled together and de novo assembled with a minimum transcript length of 200 bp for all three assemblies: cultivated+wild-assembly (CWA, including all 63 genotypes), cultivated-assembly (CA, including only cultivated genotypes), and wild-assembly (WA, including only wild genotypes). Contaminated transcripts were checked using the stand-alone DeconSeq toolkit (version 0.4.3) [29] with the default parameters. Bacterial genomes, viral genomes and human genomes downloaded from NCBI were used to build the ‘remove’ databases which were used to identify contaminant transcripts. Plant genomes of maize, rice, wheat, soybean (*Glycine max*), sorghum (*Sorghum bicolor*), *Arabidopsis thaliana*, *Brachypodium distachyon* and *Medicago truncatula* were downloaded from EnsemblPlants database and used to build the ‘retain’ databases.

### Identification of novel transcripts not present in Morex

Transcripts from the three assemblies were aligned to the Morex genome sequences (the international reference genome for barley) using the GMAP programme [30]. They were then mapped against the cDNAs (ASM32608V1.31 from EnsemblPlants database), high-confidence (HC) + low-confidence (LC) transcripts and HC + LC genes of Morex [12] using Blastn (version 2.2.28+). Transcripts with similarity < 85% and coverage < 85% were retrieved and defined as novel. They were then clustered using the cd-hit-est programme from cd-hit package v4.6.4 [31] based on sequence similarity (c = 0.95).

### Functional annotation of transcripts

ORFs (open reading frames) of transcripts were predicted using TransDecoder v3.0.0 (https://github.com/TransDecoder/TransDecoder/releases) with a minimum length of 300 nt (100 amino acids). Redundant CDS (coding DNA sequence) were removed using the cd-hit-est program (c = 0.98). For CDS from the novel transcripts, non-redundant CDS were aligned back to the reference sequences of Morex and barley Unigene database (ftp://ftp.ncbi.nih.gov/repository/UniGene/Hordeum_vulgare/), and CDS without significant hits (similarity < 85% and coverage < 85%, E-value≤1e-6) were retained. GO (gene ontology) classification for biological process was conducted by searching against plant proteins databases using the AgBase Goanna programme and summarized by GOSlimViewer with the default parameters [32]. GO enrichment analysis was performed with agriGov2.0 [33] and REVIGO [34]. HMMER v3.1b2 software [35] was used to detect Pfam-A domains (Pfam31.0) with E-value≤1e-3 [36]. NLR-parser [37] was applied to predict the NLR (nucleotide-binding leucine-rich repeat)-associated motifs and detect the NBS-LRR (nucleotide-binding site leucine-rich repeat) type disease resistance genes.

Those novel transcripts without any predicted ORFs were retrieved. They were aligned against the barley EST database (expressed sequence tag, B-EST, v2.1), full-length cDNAs of cv. *Haruna Nijo* [38] and barley UniGene database using Blastn with a minimum similarity of 90% and coverage 90% (E-value ≤1e-6). The remaining unaligned transcripts were queried against proteins from the UniProtkb_*Viridiplantae* database (EMBL-EBI) and the AgBase plant protein database [32]. Alignments with a minimum identity and coverage both at 70% were considered as significant matches (E-value ≤1e-6). Those transcripts without any significant hits to the protein databases were aligned against ncRNA sequences from the NONCODE database [39] and long ncRNAs of Morex [12] using Blastn (E-value ≤1e-6) and the Rfam database [40] with the Infernal software version 1.1.2 [41].

### SNP discovery

Trimmed reads of the representative RNA-seq datasets (dataset with the largest reads number when there are replicates for a specific accession) for each genotype were mapped to the pan-transcriptome (CWA) with Bowtie2 (v2.2.9) [42]. Redundant sequences of the CWA were firstly removed using cd-hit-est program (c = 0.95) before reference building. Duplicated reads were removed using the samtools v1.3.1 from the SAMtools package [43]. SNPs (single nucleotide polymotphisms) or SNVs (single nucleotide variants) were called by using ‘samtools mileup’ and ‘bcftools call’ [43] commands with MAPQ ≥20 and were filtered with bcftools (−e ‘DP < 4’ --SnpGap 3). The variant rates for cultivated and wild barley genotypes were annotated with SnpEff v 4.3 t [44]. Based on the gmap results with the Morex genome, SNP density along each chromosome was calculated with a 10 Mb window size. Based on SNP data from 63 cultivated and wild barley genotypes, the principle component analysis (PCA) was conducted using vcftools v0.1.14 [45] (converting the SNP vcf files to .ped and .map files), plink v1.90 beta [46] (converting .ped and .map files to binary files) and GCTA v1.91.7 beta [47] (outputting the .eigenval and .eigenvec files). The first two principal components were selected for categorizing the cultivated and wild barley genotypes.

### Genetic differentiation between cultivated and wild barley

To estimate the genetic differentiation of cultivated and wild barley, the patterns of allele frequency for each locus were measured using the SNP data from the two groups of genotypes. Gene differentiation was measured by the fixation index (F_*ST*_) using vcftools v0.1.14 [45]. Transcripts with F_*ST*_ larger than the 95th percentile were treated as having been subjected to strong selective pressures. Distribution of such transcripts on each chromosome was investigated by alignment them against the Morex genome. The KEGG (Kyoto Encyclopedia of Genes and Genomes) internal annotation tool BlastKOALA (www.kegg.jp/blastkoala/) and KAAS (https://www.genome.jp/tools/kaas/) were used to assign K numbers to those transcripts and enriched pathways were identified by KEGG mapping (www.kegg.jp/kegg/mapper.html).

We also analysed the barley *Mla* (Mildew resistance locus a) gene families which are specific to resistance to powdery mildew and genetic variations of the *Mla* locus found among cultivars [48]. Since the *Mla* locus on chromosome 1H has been subjected to extreme functional diversification and it encodes by far the largest number of R genes [49], it was selected for this study. Thirty-one published gene sequences of this locus were blast against CWA, CA and WA (identity≥90%, coverage≥50%) to identify matched transcripts. The F_*ST*_ value the *Mla* gene transcripts from CWA was extracted and the functional domains of the top hits from CA and WA were analysed by searching against NCBI Conserved Domains Databases (CDD) [50] with default settings.

## Results

### Classification and functional analysis of novel transcripts not present in Morex

**Fig. 1:**
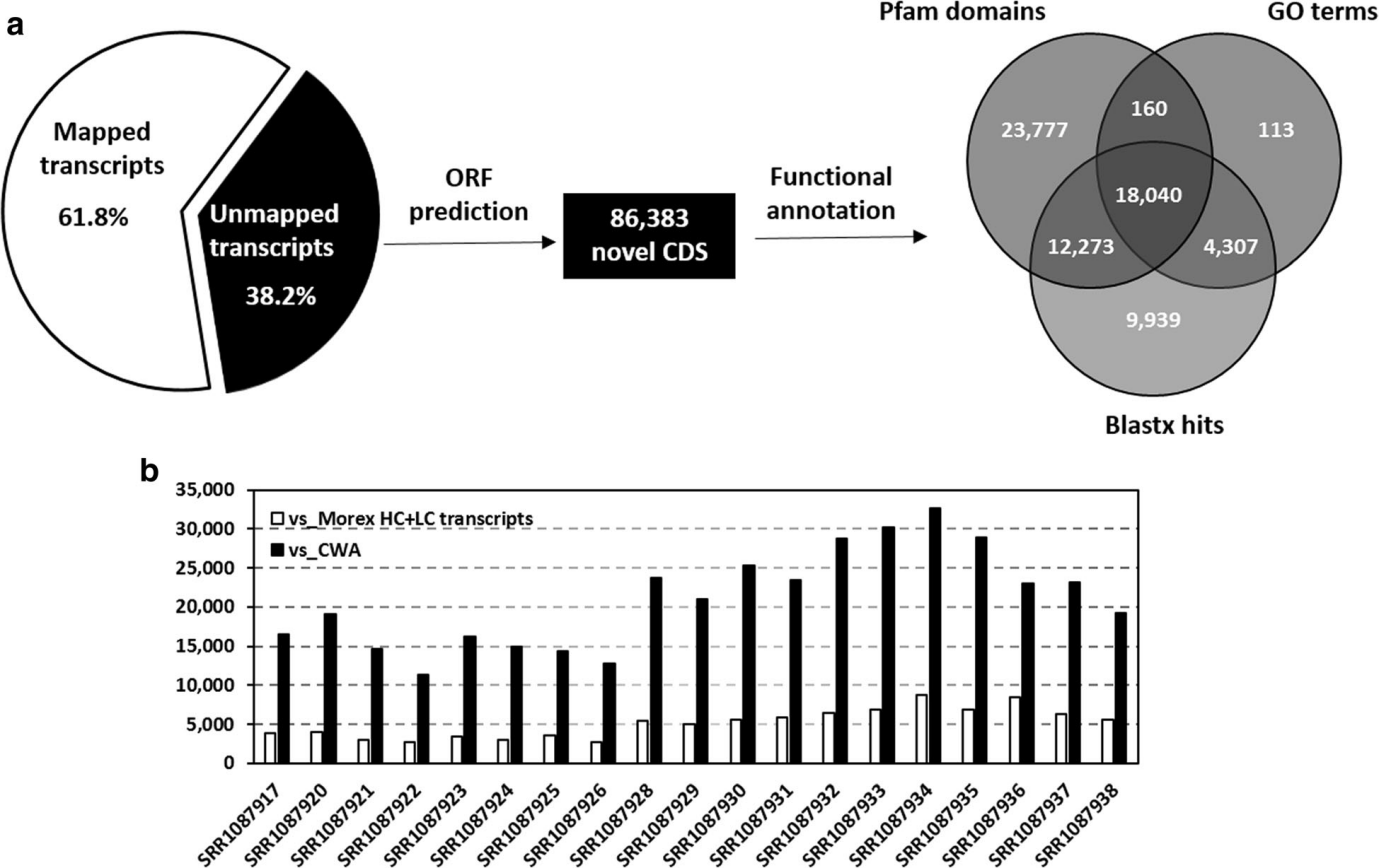
Enhanced efficiency of CWA (cultivated+wild-assembly) in detecting variations in comparison with the use of the international reference genotype Morex. **a**) Novel transcripts and CDS not detected in Morex; and **b**) difference in the numbers of SNVs detected by using either Morex HC + LC transcripts or CWA as the reference. The comparison was conducted using RNA-seq data from of 19 accessions from one study as the sequences likely have similar qualities

**Fig. 2:**
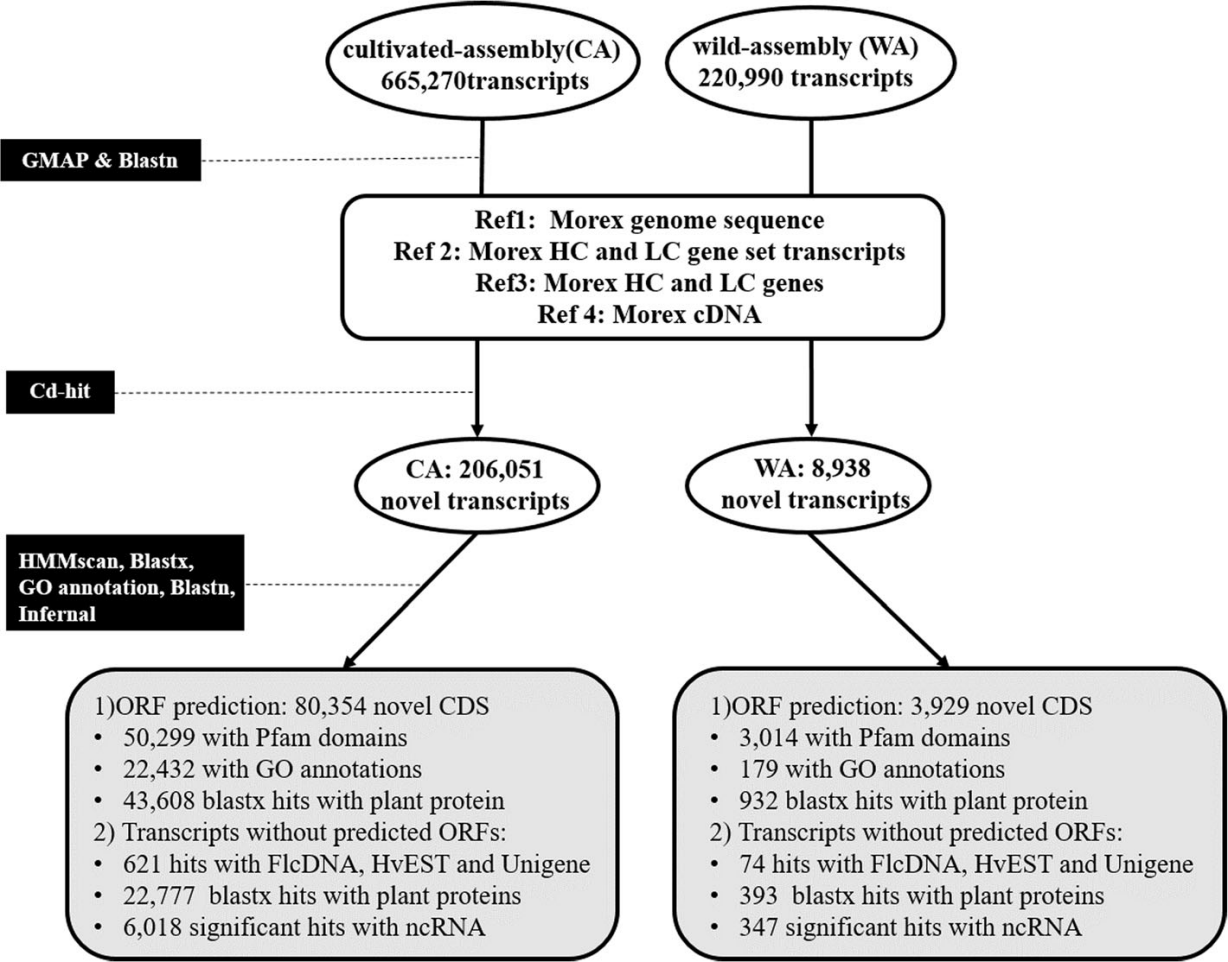
The workflow for identification and annotation of novel transcripts from cultivated and wild barley genotypes

The total number of transcripts from the cultivated+wild-assembly (CWA) were 756,632 with a N50 length of 1240 bp. When analysed against the Morex genome sequences, cDNAs, HC + LC transcripts and HC + LC genes, 289,697 of the transcripts (38.2%, with a total length of 203.9Mbp) were identified as novel (Fig. 1a). Following the removal of the redundant sequences (identity threshold 95%), 235,887 representative (non-redundant) transcripts were retained. Of the novel CDS, 86,383 were identified with predicted open reading frames (ORFs) with a minimum of 300 nt (100 amino acids). Aligning the coded protein sequences to the Pfam database identified 54,090 CDS with Pfam domains (Fig. 1a). Numbers of top assigned domains and domains related to environmental stresses were counted (Additional file 3: Figure S1). Compared with genes in Morex, 15 types of protein domains were substantially enriched (percentage difference ≥ 1.5 times) in the novel CDS. They include peptidase, LRR domain, ABC transporter, PPR (pentatricopeptide repeat) domain, short-chain dehydrogenase, exchangers/symporters and salt stress/antifungal family proteins. Meanwhile, significant hits with known plant proteins were found for 44,559 of the novel CDS, and GO annotations were assigned to 22,620 of them (Fig. 1a). GO classification for biological processes indicates a large proportion of the novel CDS was involved with ‘response to biotic and abiotic stimuli’ and ‘defense response’. These GO terms were all significantly enriched in comparison with the genes in Morex (*P < 0.05*) (Fig. 2). Aligning those transcripts without predicted ORFs against the barley EST database, the full-length cDNAs of cv. *Haruna Nijo* and the barley UniGene database found 545 transcripts with significant hits. A total of 23,556 of the remaining transcripts had significant hits to known protein databases. For those transcripts without significant hits to known proteins, 7018 detected significant matches with ncRNA (non-coding RNA) sequences.

To evaluate its efficiency in detecting genetic variations, the CWA (containing 528,646 transcripts after redundancy removal) were used as the reference for reads mapping. Trimmed RNA-seq reads of 19 cultivated and wild genotypes from a single study [20] were used for this assessment. The reads were mapped back to Morex HC + LC transcripts and CWA, respectively. Compared with the use of the Morex HC + LC transcripts (SNVs ranging from 2655 to 8753), approximately four times more SNVs were detected when CWA (SNV number ranging from 11,408 to 32,610) was used as the reference. The mapping percentage of the reads also increased from 95.9 to 98.5% when CWA was used as the reference (Fig. 1b; Additional file 4: Table S3).

### Functional comparison of transcripts from CA and WA

A total of 665,270 and 220,990 transcripts were assembled for cultivated (CA) and wild barley (WA), respectively. Of them, a total of 80,354 novel CDS for CA and 3929 for WA were identified (Fig. 2). We also found ~ 60% transcripts of CA are absent in WA and ~ 16% transcripts of WA are not present in CA. The large difference in the number of novel CDS and the proportion of common transcripts identified between cultivated and wild barley is likely due to the less diverse tissue types used (Additional file 1: Table S1) and the much smaller quantity of sequences (~157Gb compared with ~796Gb for cultivated barley) used for the wild barley genotypes. Classification of GO terms in biological process suggested a large number of the novel CDS from the wild barley were involved in ‘response to stress’, ‘multicellular organism development’, ‘cellular component organization’, ‘post embryonic development’, ‘reproduction’ and ‘anatomical structure morphogenesis’, ‘growth’, ‘cell differentiation and cell growth’. These GO terms all had substantially higher percentages (number of proteins for a specific GO term divided by the total protein number with GO terms ≥1.5 times) than those from the cultivated barley. The percentages of GO terms ‘transport’, ‘biosynthetic process’, ‘embryo/flower development’, ‘lipid/carbohydrate/secondary metabolic process’ and ‘regulation of gene expression’ are substantially higher for novel CDS from the cultivated barley.

**Fig. 3:**
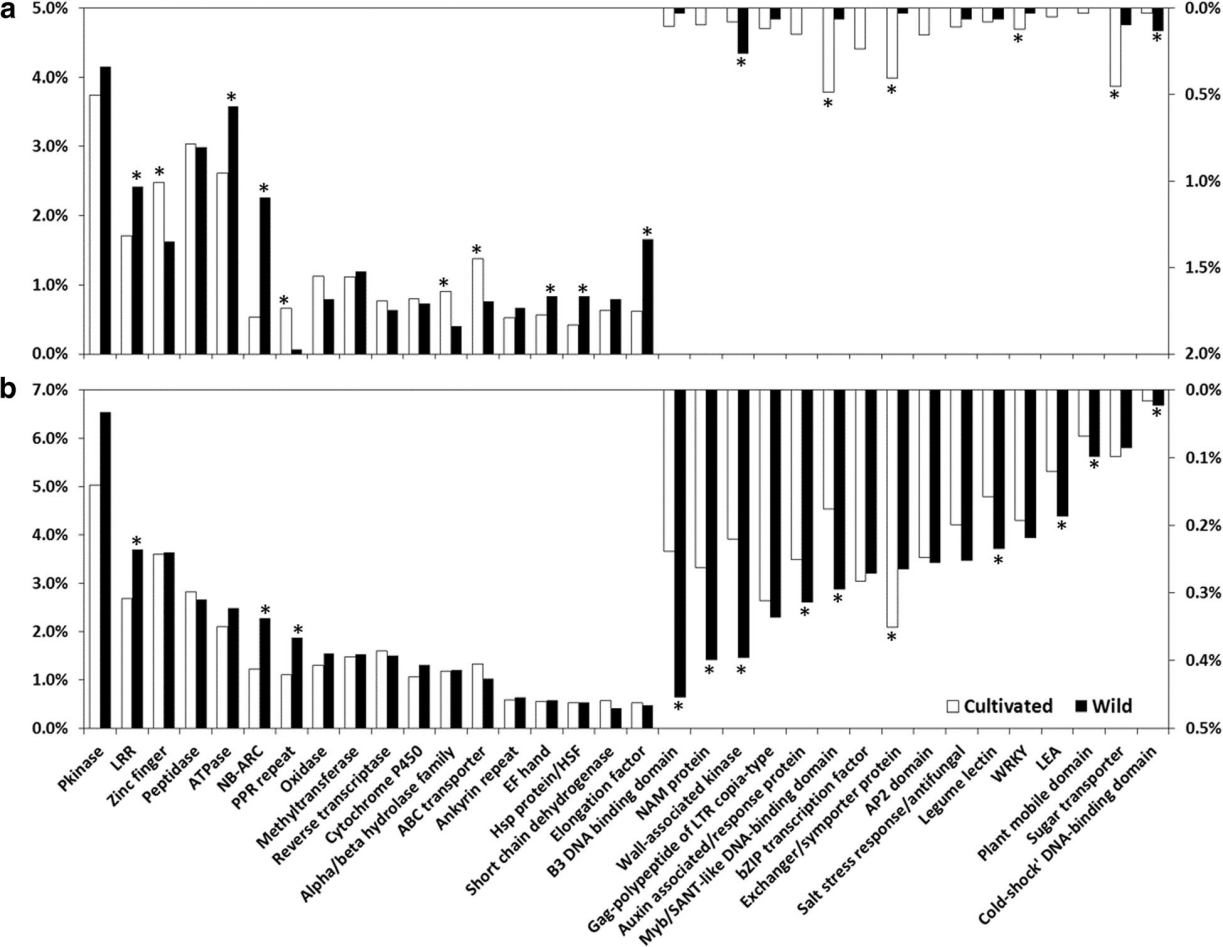
Difference in annotated protein domains between cultivated and wild barley. **a** Difference in the numbers of novel CDS. **b** Difference between all predicted CDS. The Y-axis shows the percentage of CDS with a specific protein domain. Protein domains marked with * indicate significant difference between cultivated and wild barley

Pfam domains were assigned to 50,299 of the novel CDS from the cultivated and 3014 of those from the wild barley genotypes (Fig. 3). The proportions of several protein classes, including LRR, NB-ARC (nucleotide-binding adaptor shared by APAF-1, R proteins, and CED-4), elongation factor, HSP (heat shock protein) /HSF (heat shock factor) protein, EF-hand, wall-associated kinase (WAK) and ‘cold-shock’ DNA-binding domain-containing proteins, are more than 1.5 times higher in the wild barley than those in cultivated genotypes (Fig. 3a). Similar analysis of CDS from all transcripts of CA and WA showed that the genes with LRR, NB-ARC, WAK, Myb/SANT (SWI3, ADA2, N-CoR, and TFIIIB)-like DNA-binding domain, LEA (late embryogenesis abundant) and plant mobile domains are more abundant in wild barley genotypes than those of cultivated ones (Fig. 3b). Apart from the Pfam annotation, the NLR-parser analysis also showed that both novel CDS and the whole CDS set of wild barley had a significantly higher proportion (two to five fold higher) of NLR genes (including both CNL and TNL genes) than those of cultivated barley (Table 1).

**Table 1:** Difference in NLR genes identified between cultivated and wild barley with NLR-parser analysis

CDS sets	CC^a^-NBS-LRR(CNL)	TIR^b^-NBS-LRR(TNL)	Number of NLR genes	Number of total CDS/genes	Percentage of NLR genes
novel CDS of CA	114	58	172	80,354	0.21%
novel CDS of WA	39	1	40	3929	1.02%
all CDS of CA	669	66	735	134,773	0.55%
all CDS of WA	493	10	503	50,680	0.99%

^a^CC = coiled coil; ^b^TIR = Toll/Interleukin1 receptor

### Genetic diversity of wild barley and cultivated barley with SNP discovery

Using CWA as reference, a total of 441,770 SNPs were identified among the cultivated barley genotypes and 672,579 SNPs were detected among the wild barley genotypes. The number (485, 473) and proportion (72.2%) of unique SNPs were significantly higher in the wild barley genotypes than those in the cultivated ones (254,664, 57.6%). SnpEff annotation analysis also found higher genetic diversity among the wild barley genotypes (1/193, one variant for every 193 bases) than that among the cultivated ones (1/144). The distribution of SNPs along each of the chromosomes is visualized with a 10 Mb window size (Additional file 5: Figure S2). A typical V-shaped distribution pattern was observed for all chromosomes. Chromosome 7H shows the largest difference in SNP density (with an average of 1.8 times) between cultivated and wild barley genotypes. PCA analysis indicated the SNP data can effectively discriminate the cultivated and wild barley. They were clustered into two distinct groups with no obvious overlapping although five of the cultivated barley accessions show large genetic distances from the others (Additional file 6: Figure S3).

### Disease resistance genes suffered more selective pressures during barley domestication

**Fig. 4:**
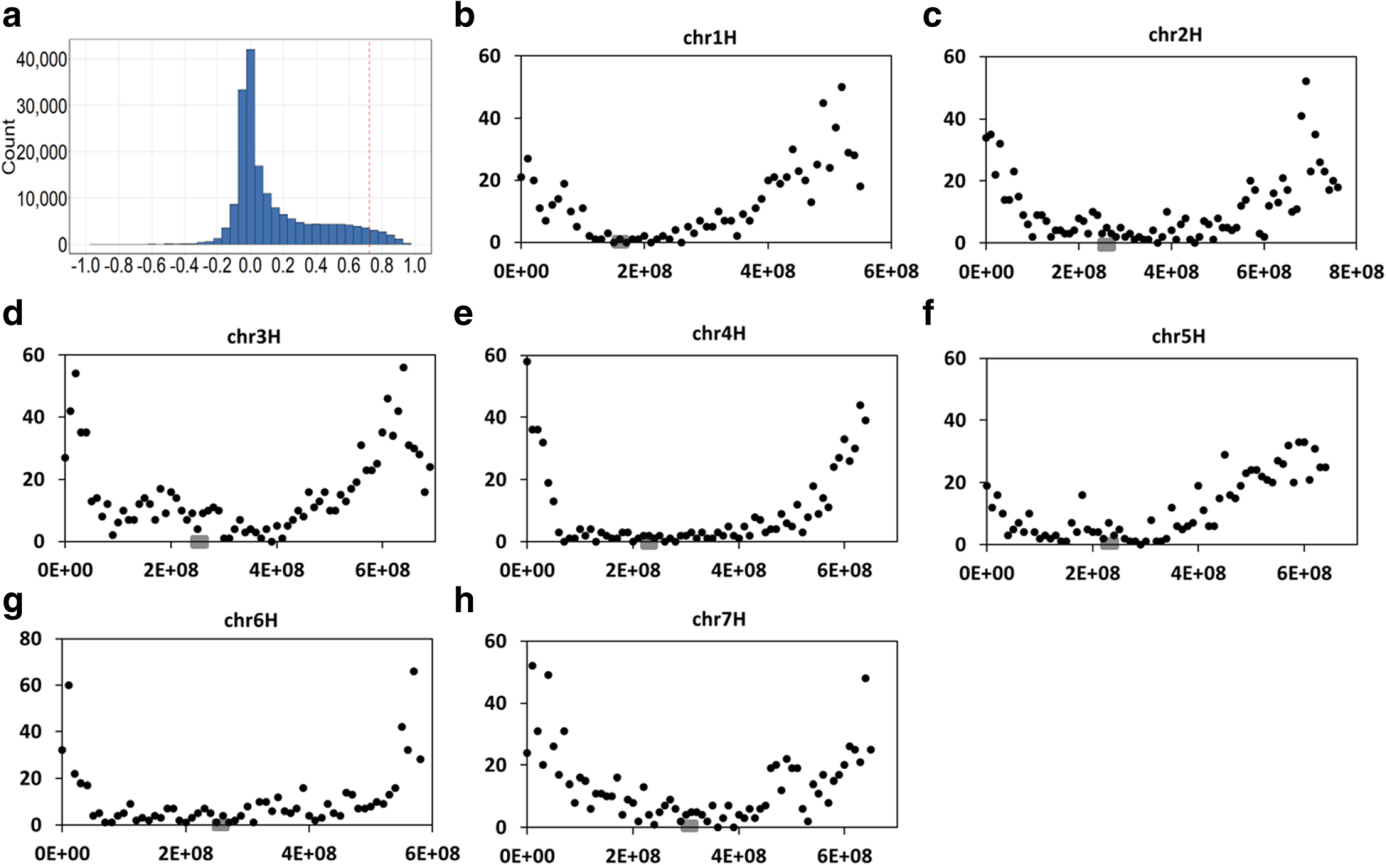
Overview of genetic differentiation between cultivated and wild barley with measured *F*_*ST*_. Physical locations of transcripts from CWA were based on gmap results with the Morex genome assembly. **a** Frequency distribution of *F*_*ST*_ value for each locus. The X-axis indicates the value of *F*_*ST*_. The red dash vertical line indicates the threshold at 0.72 (the 95th percentile threshold)*.* **b-h** Chromosomal distributions of transcripts with *F*_*ST*_ larger than 0.72 with a window size 10 Mb. The X-axis indicates the physical positions of each chromosome and Y-axis shows the number of transcripts. The grey boxes on Y-axis indicates the approximate location of the centromere for each of the chromosomes

**Fig. 5:**
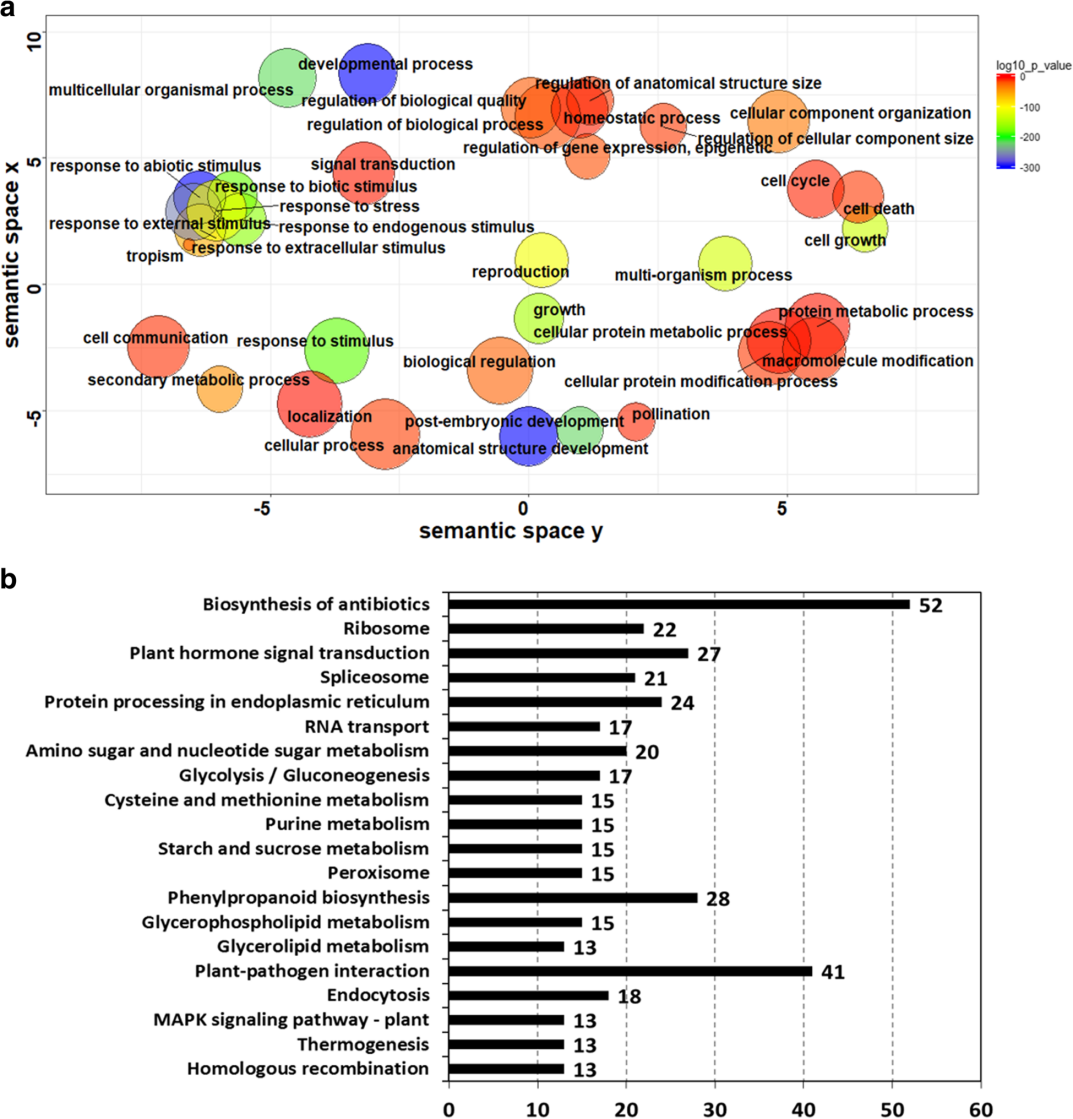
Functional annotation of genes under strong selective pressures (F_*ST*_ > 0.72). **a** GO enrichment analysis; (**b**) count of genes (X-axis) in top 20 enriched KEGG pathways excluding the two global/overview maps (metabolic pathways and biosynthesis of secondary metabolites pathway)

With the 95th percentile of F_*ST*_ values (0.72) as the threshold, a total of 6520 transcripts were found under strong selection pressures (Fig. 4a). These transcripts are distributed unevenly between different chromosomes although the ‘V-shaped’ distribution is conserved for all chromosomes (Fig. 4b-h). Chromosome 3H possesses the largest number of genes under strong selection while there is little difference between the other six chromosomes. GO enrichment analysis on the 3165 genes predicted from these transcripts found that they are heavily involved in biological processes including ‘response to stress’, ‘response to abiotic/biotic stimulus’, ‘development process’, ‘anatomical structure development’, ‘regulation of biological process’ and ‘secondary metabolic process’ (Fig. 5a). When analysed against the KEGG pathway database, these genes were found to be enriched in pathways of biosynthesis of antibiotics, plant-pathogen interaction, phenylpropanoid biosynthesis and plant hormone signal transduction (Fig. 5b).

The blast analysis of the 31 variants of *Mla* locus against CWA identified eight matched transcripts and only two of them had F_*ST*_ values: 0.49 for TR120931_c5_g1_i1and 0.53 for TR120931_c5_g1_i6. This indicate the *Mla* gene families have still been under high selection pressure. When separating cultivated and wild barley genotypes, the top CA hits of *Mla* genes are TR100774_c1_g1_(i2, i3, i5, i6,i7), all of which have ≥90% identity and 100% query coverage. The average sequence length of these transcripts is 3687 bp. The best matched transcripts from WA are TR133441_c5_g1_(i1, i6) with a maximum of 66% query coverage. The CDD analysis found that TR100774_c1_g1_i6 of CA (3752 bp, the longest *Mla* gene transcript of CA) has CC and NB-ARC domains and 5 LRR repeats while the transcript TR133441_c5_g1_i1 (2160 bp, the longest *Mla* gene transcripts of WA) has only CC and NB-ARC domains without any predicted LRR repeats (Additional file 7: Figure S4).

## Discussion

In the study reported here we constructed a pan-transcriptome of barley by de novo assembling 288 sets of RNA-seq data from 63 genotypes. Approximately 38.2% of the transcripts from the newly assembled pan-transcriptome were not found in the genome of Morex. The novel transcripts were enriched with genes associated with response to abiotic and biotic stresses. Comparing the cultivated and wild barley genotypes at the pan-transcriptome level found that disease resistance genes are more abundant in the wild barley and they have suffered stronger selective pressures during barley domestication in comparison with genes in other categories.

As a proportion of genes in Morex must also belong to the dispensable genome component, it seems not unreasonable to speculate that the ratio of dispensable genome in barley could be even higher than 38.2%. However, it is unlikely that all genes from all of genotypes were captured in the RNA-seq data used in this study. Incomplete capture of all genes in individual genotypes invariably lead to an exaggerated proportion of the dispensable genome. Nevertheless, considering the large numbers of genotypes and sequences used in this study, the pan-transcriptome has likely captured the majority of the expressed genes in this species. The proportion of the dispensable genome found here for barley is similar to that for bread wheat [6, 7]. With the addition of such a large number of novel transcripts on top of those from Morex, the newly assembled pan-transcriptome should facilitate various investigation in barley and its close relatives. We demonstrated as part of this study that, compared with the use of Morex as the reference, four times more SNVs were detected when CWA was used (Fig. 1b).

Compared with the cultivated genotypes, wild barley contains substantially more disease resistance genes. This is the case for both the whole transcripts and novel ones only (Fig. 3). Although a larger number of novel CDS are identified from CA than those from WA, the difference unlikely contributed significantly to the comparison as it was conducted using the proportion values. The disease resistance genes include those with NB-ARC, LRR and WAK domains [51–54]. It has been reported that the LRR receptors and WAK are not only regulating plant immunity but also tightly linked to other yield-related genes [55]. NLR-parser analysis also revealed that the wild barley genotypes contained higher percentage of genes conferring disease resistance in comparison with the cultivated ones (Table 1). Importantly, the difference does not seem to be caused by how the samples used in generating the RNA-seq data were obtained. Although the ratio of the disease resistance genes was higher in the wild barley genotypes than the cultivated ones, none of the RNA-seq datasets from the former were generated from plants challenged with any biotic pathogen while 16 datasets from cultivated barley genotypes were generated from tissues infected by spider mite (Additional file 1: Table S1). Therefore, the observed difference must, to a large degree, reflect genuine differences between cultivated and wild barley. The genetic differentiation analysis also found that the enriched genes under strong selective pressures are mainly those involved in ‘response to stress’, ‘plant-pathogen interaction’, ‘phenylpropanoids biosynthesis’ and ‘plant hormone signal transduction’ (Fig. 5). It has been reported the phenylpropanoids can contribute to plant responses towards biotic stimuli and plant hormones can act as signals to trigger defense responses [56, 57]. Thus, these two enriched pathways may help explain why wild barley expresses a higher proportion of disease resistance genes even without pathogen infection. Moreover, the results from the analysis of the *Mla* gene transcripts provide also further evidence showing that genes related to disease resistance have suffered stronger selection than other gene categories during domestication.

As the increased yield potential is one of the most important changes following domestication and breeding [58], the substantially reduced numbers of genes responsive to diseases in the cultivated genotypes suggest the likelihood that disease resistance can incur costs in yield potential. This likelihood is not different from previous studies showing that resistance often incur physiological costs that reduce host fitness in the absence of the disease in concern in different plant species [59, 60]. It is believed that such costs can arise because the defence strategy could have harmful pleiotropic effects [61] or because investment in defence requires allocation of limiting resources and hence trade-offs with other traits [59]. Many crop plants have also been characterised by low levels of resistance to pathogen infection [62]. The likelihood that resistance may incur costs that reduce host fitness or yield potential suggests that incorporating a large number of resistance genes into a single genotype may not be an effective breeding strategy. Rather, targeting only genes resistant to major disease for a given environment can be more efficient in breeding cultivars with high yield potential.

## Conclusions

In our study, we constructed a barley pan-transcriptome by using 63 different genotypes. At the pan-transcriptome level, we demonstrated that the disease resistance genes went through stronger selective pressures than other gene categories during barley domestication. With the trade-offs between gaining yield potential and increasing disease resistance during domestication and breeding, we infer that targeting only genes for major diseases for a given environment can be more efficient in variety breeding than incorporating all resistance genes into a specific genotype.

## Additional files

Additional file 1: **Table S1.** Summary of RNA-seq datasets used in this study. (XLSX 15 kb)

Additional file 2: **Table S2.** Different filtering criteria for raw sequences of RNA-seq data with different spot lengths (AvgSpotLen). (XLSX 9 kb)

Additional file 3: **Figure S1.** Functional annotation of novel CDS from CWA. (a) Difference in the percentage of Pfam domains between Morex and CWA (substantially enriched Pfam domain highlighted with *) and (b) Significantly enriched GO terms for biological processes in CWA in comparison with those in Morex (*P* < 0.05). (PNG 83 kb)

Additional file 4: **Table S3.** SNVs detected by mapping RNA-seq of 19 barley accessions against either Morex HC + LC or CWA. (XLSX 11 kb)

Additional file 5: **Figure S2.** Distribution of SNPs along each of the seven chromosomes (a-f). Physical locations of transcripts from CWA were based on the gmap results with the Morex genome assembly. Red line stands for cultivated barley and the blue for wild barley. X-axis shows the physical position of each chromosome and Y-axis indicates the count of SNPs. (PNG 136 kb)

Additional file 6: **Figure S3.** PCA results of the 63 genotypes based on the SNP data. (PNG 61 kb)

Additional file 7: **Figure S4.** Comparison of functional domains of the longest *Mla* gene transcript from CA with that from WA. (a) TR100774_c1_g1_i6 of CA; and (b) TR133441_c5_g1_i1of WA. (PNG 289 kb)

## Abbreviations

CA: Transcript assembly of cultivated genotypes
CC: Coiled Coil
CDD: Conserved Domains Databases
CDS: Coding DNA sequence
CNL: CC-NBS-LRR
CWA: Transcript assembly of cultivated and wild genotypes
EBI: European Bioinformatics Institute
EMBL: European Molecular Biology Lab
ENA: European Nucleotide Archive
EST: Expressed sequence tag
F_ST_: Fixation index
GEO: Gene Expression Omnibus
GO: Gene ontology
HC: High-confidence
HSF: Heat shock factor
HSP: Heat shock protein
KEGG: Kyoto Encyclopedia of Genes and Genome
LC: Low-confidence
LEA: Late embryogenesis abundant
LRR: Leucine-rich repeat
Mla: Mildew resistance locus a
NB-ARC: Nucleotide-Binding adaptor shared by APAF-1, R proteins, and CED-4
NBS: Nucleotide-binding site
NCBI: National Center for Biotechnology Information
ncRNA: Non-coding RNA
NLR: Nucleotide-binding leucine-rich repeat
ORF: Open reading frame
PCA: Principle component analysis
PPR: Pentatricopeptide repeat
SANT: SWI3, ADA2, N-CoR, and TFIIIB
SNP: Single nucleotide polymorphism
SNV: Single nucleotide variants
SRA: Short Sequence Read Archive
TIR: Toll/Interleukin1 receptor
TNL: TIR-NBS-LRR
WA: Transcript assembly of wild genotypes
WAK: Wall-associated kinase
