# Supplementary figures and images for: A pan-transcriptome analysis shows that disease resistance genes have undergone more selection pressure during barley domestication
Source: BMC Genomics. 2019 Jan 7;20:12. doi: 10.1186/s12864-018-5357-7 (PMC6323845; doi:10.1186/s12864-018-5357-7)

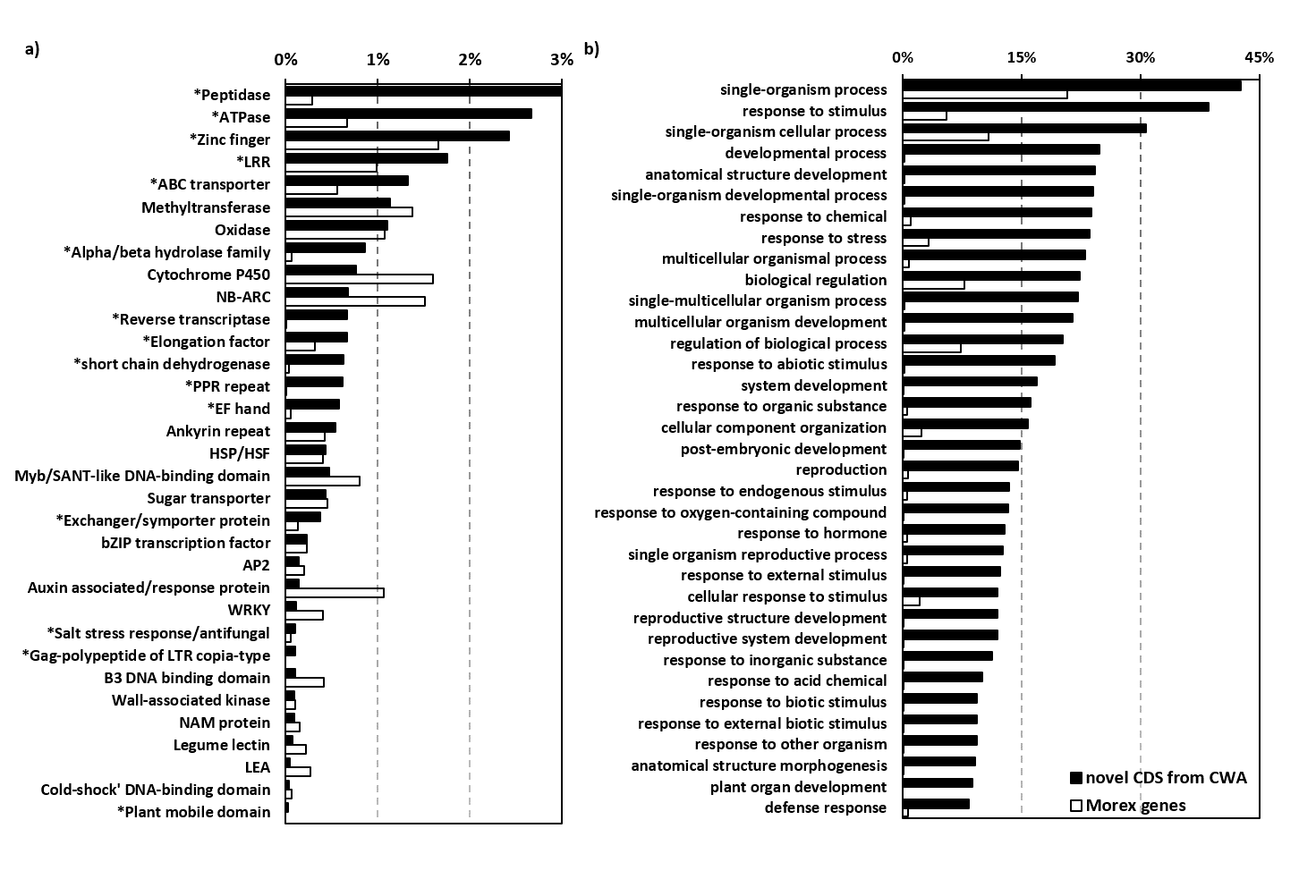

Supplement: Supplementary file 3 — Figure S1. Functional annotation of novel CDS from CWA. (a) Difference in the percentage of Pfam domains between Morex and CWA (substantially enriched Pfam domain highlighted with *) and (b) Significantly enriched GO terms for biological processes in CWA in comparison with those in Morex (P < 0.05). (PNG 83 kb) [file 12864_2018_5357_MOESM3_ESM.png]

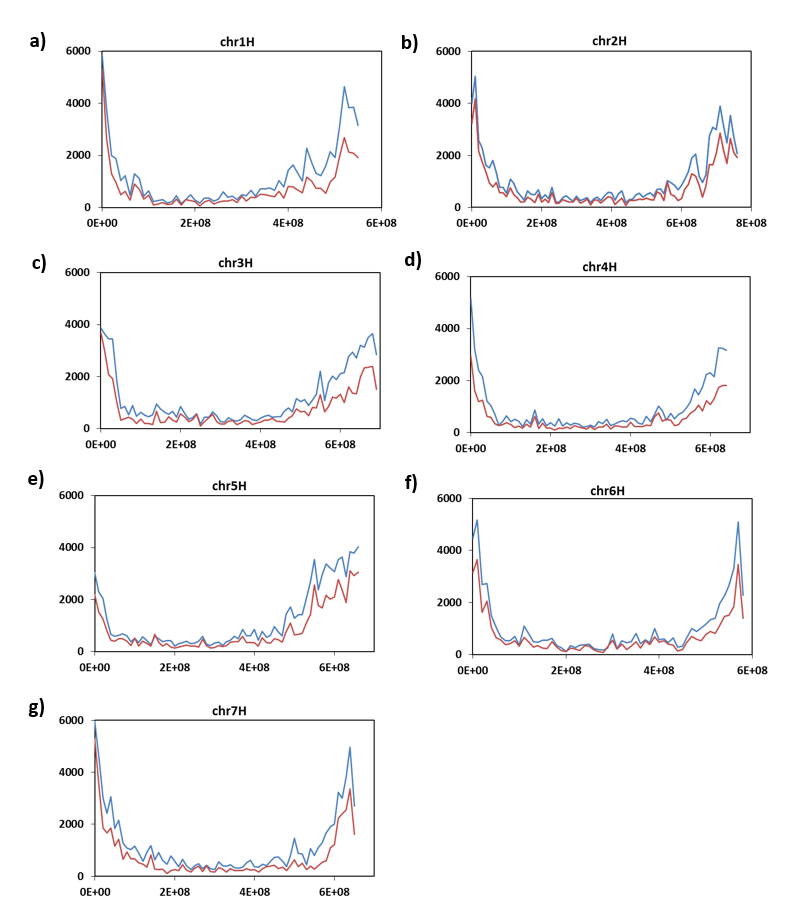

Supplement: Supplementary file 5 — Figure S2. Distribution of SNPs along each of the seven chromosomes (a-f). Physical locations of transcripts from CWA were based on the gmap results with the Morex genome assembly. Red line stands for cultivated barley and the blue for wild barley. X-axis shows the physical position of each chromosome and Y-axis indicates the count of SNPs. (PNG 136 kb) [file 12864_2018_5357_MOESM5_ESM.png]

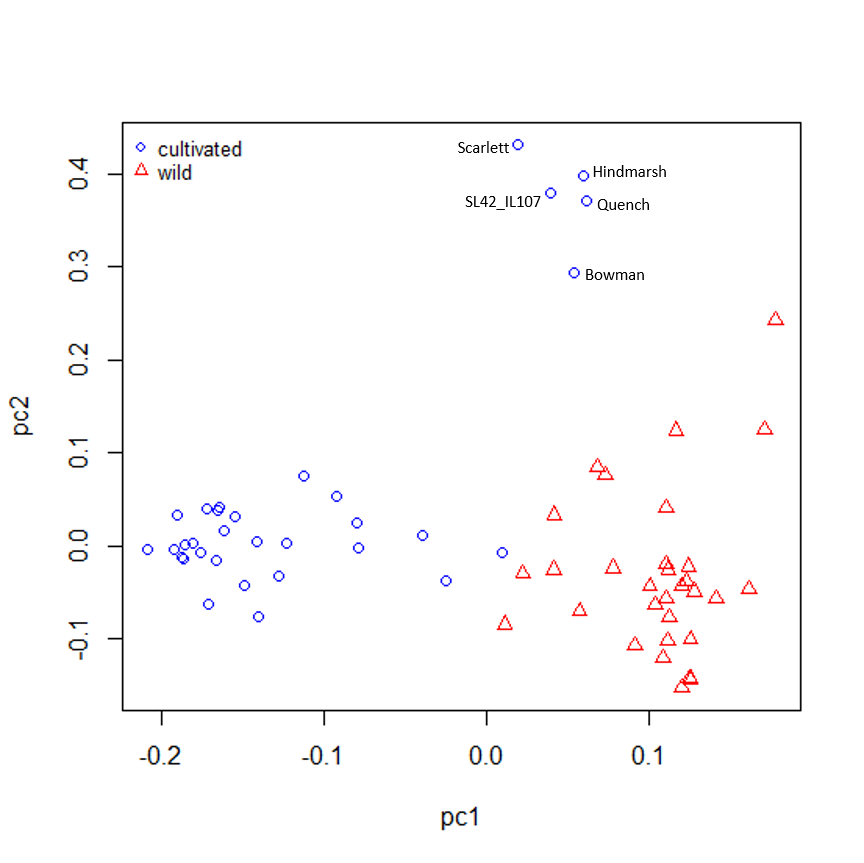

Supplement: Supplementary file 6 — Figure S3. PCA results of the 63 genotypes based on the SNP data. (PNG 61 kb) [file 12864_2018_5357_MOESM6_ESM.png]

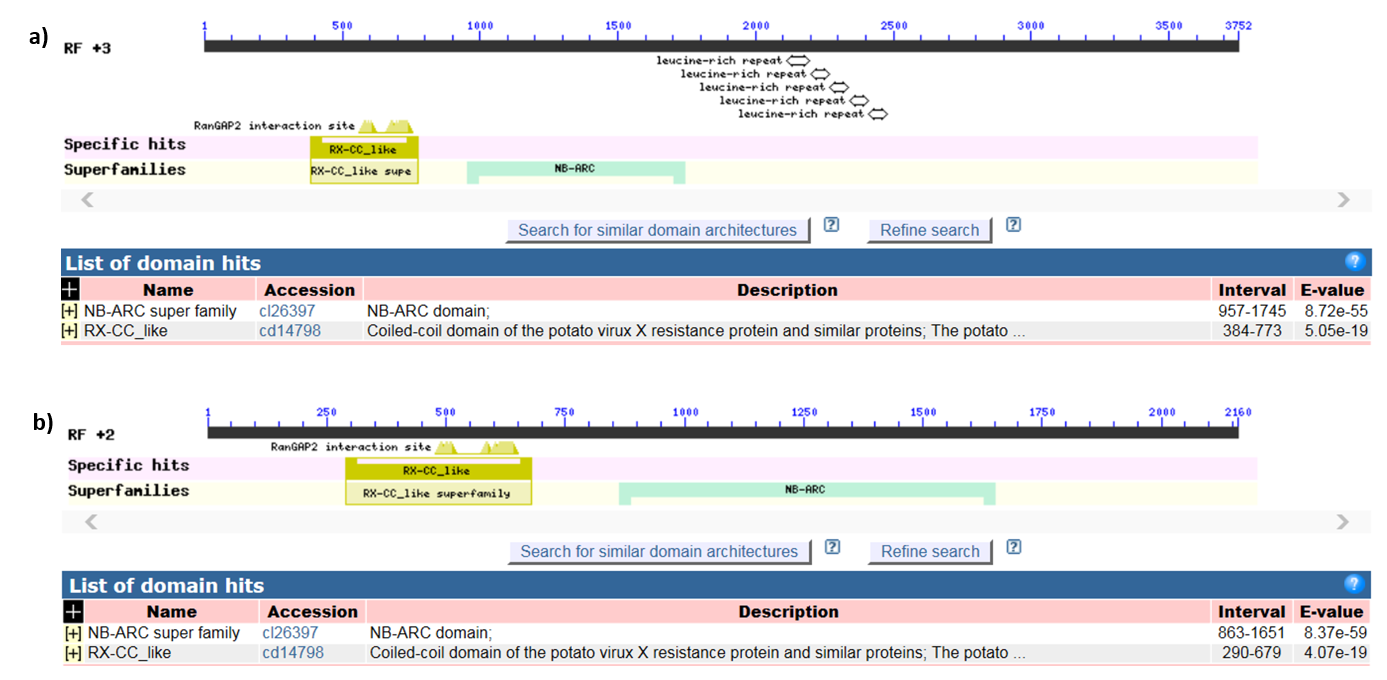

Supplement: Supplementary file 7 — Figure S4. Comparison of functional domains of the longest Mla gene transcript from CA with that from WA. (a) TR100774_c1_g1_i6 of CA; and (b) TR133441_c5_g1_i1of WA. (PNG 289 kb) [file 12864_2018_5357_MOESM7_ESM.png]
